# It All Starts at the Ends: Multifaceted Involvement of C- and N-Terminally Modified Cholinesterases in Alzheimer’s Disease

**DOI:** 10.5041/RMMJ.10014

**Published:** 2010-10-31

**Authors:** Amit Berson, Hermona Soreq

**Affiliations:** Department of Biological Chemistry and the Edmond and Lily Safra Center of Neuroscience, The Hebrew University of Jerusalem, Jerusalem, Israel

**Keywords:** Acetylcholinesterase, Alzheimer’s disease, apoptosis, beta-amyloid, butyrylcholinesterase

## Abstract

In Alzheimer’s disease (AD), premature demise of acetylcholine-producing neurons and the consequent decline of cholinergic transmission associate with the prominent cognitive impairments of affected individuals. However, the enzymatic activities of acetylcholinesterase (AChE) and butyrylcholinesterase (BChE) are altered rather late in the disease progress. This raised questions regarding the causal involvement of AChE and BChE in AD. Importantly, single nucleotide polymorphisms (SNPs), alternative splicing, and alternate promoter usage generate complex expression of combinatorial cholinesterase (ChE) variants, which called for testing the roles of specific variants in AD pathogenesis. We found accelerated amyloid fibril formation in engineered mice with enforced over-expression of the AChE-S splice variant which includes a helical C-terminus. In contrast, the AChE-R variant, which includes a naturally unfolded C-terminus, attenuated the oligomerization of amyloid fibrils and reduced amyloid plaque formation and toxicity. An extended N-terminus generated by an upstream promoter enhanced the damage caused by N-AChE-S, which in cell cultures induced caspases and GSK3 activation, tau hyperphosphorylation, and apoptosis. In the post-mortem AD brain, we found reduced levels of the neuroprotective AChE-R and increased levels of the neurotoxic N-AChE-S, suggesting bimodal contribution to AD progress. Finally, local unwinding of the α-helical C-terminal BChE peptide and loss of function of the pivotal tryptophan at its position 541 impair amyloid fibril attenuation by the common BChE-K variant carrying the A539T substitution, *in vitro*. Together, our results point to causal yet diverse involvement of the different ChEs in the early stages of AD pathogenesis. Harnessing the neuroprotective variants while reducing the levels of damaging ones may hence underlie the development of novel therapeutics.

Alzheimer’s disease (AD), the leading cause of dementia in the elderly today, is a neurodegen-erative disorder with an urgent and unmet medical need.^1^–^3^ AD is characterized by several hall-marks including increased levels of amyloid beta 42 (Aβ42) and the consequent generation of toxic oligomers and plaques, intracellular accumulation of neurofibrillary tangles composed of hyperphosphorylated tau protein, synaptic deficits, and neuronal loss.^1^–^3^ The currently used cholinesterase inhibitor therapies mainly offer palliative relief, and a thorough understanding of the early stages of the disease is needed for successful future interventions. Interestingly, both acetylcholinesterase (AChE) and butyrylcholinesterase (BChE) are localized in amyloid plaques, and early reports showed that AChE is capable of facilitating Aβ fibril formation.^4^ However, AChE is not one but several enzymes generated by alternate promoter usage and alternative splicing (Figure 1).^5^,^6^ At the C-terminus, skipping of exon 5 and inclusion of exon 6 generates the normally abundant AChE-S variant which includes a helical C-terminus^7^ and is attached as tetramers through a dedicated structural unit to the synaptic cleft.^8^ Inclusion of in-frame intron 4 and exon 5 generates the stress-induced monomeric and soluble AChE-R variant with its naturally unfolded C-terminal peptide.^9^ AChE has been attributed roles in apoptosome formation and apoptosis.^10^ However, it remained unclear if different variants participate in this function similarly. Also, AChE is widely expressed in the healthy brain; it therefore remained unclear whether it plays similar role(s) in the apoptotic pathway in the healthy brain and in AD. Based on these arguments, we reasoned that differential expression of AChE variants may be causally involved in the pattern of neuronal death seen in AD. To address the specific functions of these variants in AD we first compared the effect of recombinant, highly purified AChE-S and AChE-R on amyloid β-sheet formation using thioflavin-T incorporation assay. While AChE-S facilitated the formation of β-sheets, AChE-R surprisingly inhibited oligomerization and β-sheet formation. The neurotoxic amyloid peptides Aβ40 and Aβ42 were dose-dependently modulated by the two variants, and masking the C-terminus of AChE-R using a specific antibody blocked this effect.^11^

**Figure 1:**
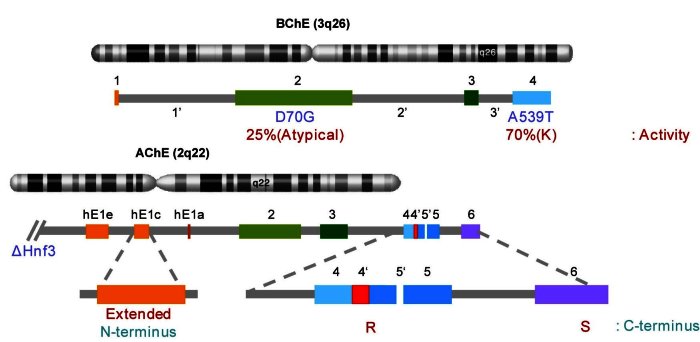
Genomic location and gene structure of BChE and AChE. Both enzymes encode many different variants, BChE due to multiple polymorphisms and AChE because of alternate promoter usage and 3’ alternative splicing. Noted are those single nucleotide polymorphisms in BChE which generate the two most frequent variants in Middle Eastern populations, the C-terminal A539T substitution in BChE-K and the N-terminal D70G mutation in “atypical” BChE which causes post-anesthetic depression and is linked to apnea.^43^ BChE-K retains approximately 70% of its hydrolytic activity due to inherent instability, whereas “atypical” BChE retains only 25% of this activity. In AChE, alternate promoters generate N-terminally extended variants, and alternative splicing changes the C-terminus of the protein.^39^

Several neurotoxic Aβ oligomers have thus far been described,^12^,^13^ and while it is generally believed that soluble Aβ is the main toxic species in AD,^2^ insoluble amyloid plaques also induce damage to dendrites and disrupt normal neuronal wiring.^14^ For these reasons the *in-vivo* effect of AChE-S and AChE-R was examined in the APPsw mouse model, carrying the amyloid precursor protein (APP) with the “Swedish” mutation leading to early-onset Alzheimer’s disease. Two missense mutations in APP result in these mice in increased Aβ42/40 ratio, amyloid plaque formation, synaptic deficits, and learning and memory impairments. To challenge the hypothesis that specific AChE variants could affect the progress of these neuropathology hall-marks, we crossed the APPsw mice with mice engineered to over-express either AChE-S or AChE-R. Similarly to the *in-vitro* results, we found that AChE-S facilitated the formation of plaques.^15^ Mice co-expressing AChE-S and APPsw showed more plaques, and these appeared earlier in the double-transgenic mice than in mice transgenic for APPsw alone. More-over, APPsw/AChE-S mice showed memory impairments that were tightly correlated with plaque burden.^16^ In contrast, AChE-R reduced the overall brain area covered with these aggregates.^11^ Secondary outcomes of amyloid toxicity were also modulated: AChE-R reduced gliosis and restored dendritic density. In parallel to the mouse studies, we further explored human brain tissues from AD patients and matched controls. Total AChE expression in the AD brain was reduced only to a minor extent, but AChE-R levels were drastically reduced to about 20% of control levels,^11^ supporting the notion that loss of this naturally rare variant may be involved with amyloid plaque development in the human brain as well.

In addition to the alternate C-terminus of AChE, changes in the composition of the N-terminus are also important for the functions and properties of this enzyme. Several alternate promoters in mouse and human AChE have been reported.^9^ Among these, of particular interest is an upstream promoter leading to an N-terminal extension via an in-frame translation start site. This N-terminal extension can be combined with either the AChE-S or AChE-R unique C-termini leading to four different variants (AChE-S, AChE-R, N-AChE-S, and N-AChE-R). Transfection of primary cortical neurons and cell lines of other tissue origins with the four variants demonstrated that the N-AChE-S variant is the only one which induces apoptosis. Further, N-AChE-S levels are increased following thapsigargin treatment which induces apoptosis by increasing intracellular calcium levels. Concomitant activation of caspases 3 and 9 was observed following N-AChE-S transfection. Moreover, the apoptotic effect of N-AChE-S was abolished by small interfering RNA (siRNA) against AChE, cholinesterase inhibitors, apoptosis inhibitors, and by transfection of the anti-apoptotic Bcl proteins.^17^,^18^ A key player in apoptosis and Alzheimer’s disease is glycogen synthase kinase 3 (GSK3).^19^,^20^ We therefore investigated whether N-AChE-S affects GSK3 activation. Indeed, reduced levels of serine-phosphorylated inactive GSK3 were observed after N-AChE-S transfection. One substrate of GSK3 is the microtubule-associated protein tau. As expected, N-AChE-S also induced tau hyperphosphorylation, and the expression pattern of these two proteins in the post-mortem cortex of AD patient donors was remarkably similar. Attempts to generate N-AChE-S transgenic mice have thus far been unsuccessful given that over-expression of N-AChE-S is lethal, with almost no embryo passing the morula stage.^18^ Given that most AD mouse models used to date do not show neuronal death, a major hall-mark of AD, combining existing models with mild N-AChE-S over-expression may lead to a new and more relevant model. To delineate the mechanism(s) of N-AChE-S-induced apoptosis we hence searched for protein partners *in vitro* and found that GSK3, the Aurora and cyclin-G-dependent kinases (GAK), membrane integrin receptors and the death receptor FAS all interact with N-AChE-S.^18^ Therefore, N-AChE-S seems to be a key factor in apoptosis, especially in the AD-related context of calcium dys-homeostasis and tau hyperphosphorylation.

Contrasting the many variants of AChE, there is no evidence that BChE transcripts undergo alternative splicing or are generated from different promoters. However, the BChE gene is considerably more susceptible to mutability than the AChE gene. Over 40 genomic variants have been described, with some of them having profound effects on the hydrolytic properties of this enzyme. Among these polymorphisms, the alanine-to-threonine substitution at position 539 is the most frequent one, with allelic frequencies of 0.13–0.21 (Figure 1). This variant, termed BChE-K, is a long-debated risk factor for AD. While several studies found that BChE-K confers high risk to develop AD,^21^ others have found no association,^22^ or even found it to be protective.^23^ We therefore took a biochemical approach and compared the influence of “usual” BChE (BChE-U) and BChE-K on amyloid oligomerization and toxicity. BChE-U was found to act similarly to AChE-R and attenuated amyloid oligomerization.^24^,^25^ This effect was mainly dependent on a tryptophan residue which disturbs an amphipathic α-helix at the C-terminus (Figure 2). The effect of BChE-K seems to be a complex one, with its reduced hydrolytic activity protecting cholinergic transmission, whereas its impaired C-terminal structure interferes with this protein’s capacity to attenuate amyloid fibril formation.^26^ Therefore, the combined effect of BChE-K may depend on other factors modulating its activity, which could explain at least some of the controversy reported in the literature.^27^

**Figure 2:**
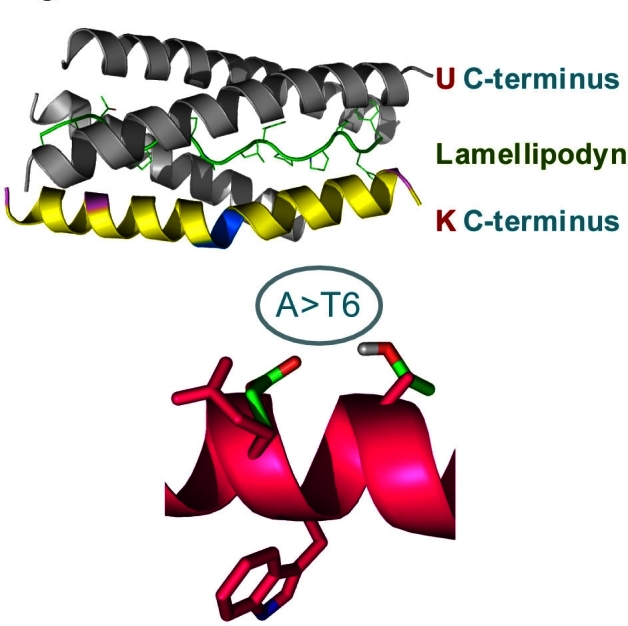
Structural effects of alanine-to-tryptophan substitution in position 539 of BChE. Shown is molecular modeling of the helical C-terminal peptides of the “usual” (wild-type) BChE (gray) and the BChE-K variant (yellow), as these interact with the proline-rich Lamellipodin peptide with which BChE is associated in the serum. Note that the A-to-T mutability, characteristic of the K variant, and which is schematically drawn below, induces a kink. This impairs protein-protein interactions of the BChE-K C-terminal peptide, possibly by changing the positioning of the adjacent tryptophan – as we could experimentally validate by nuclear magnetic resonance measurements.^26^

The cholinesterases are not unique in the different features conferred by their modified N- and C-termini; rather, many other neurodegeneration-related proteins show different and sometimes inverse features when their N- and/or C-termini are modified, either by alternative splicing or due to alternate promoter usage or single residue substitutions. Examples of functionally effective alternative splicing in the terminal regions include presenilins 1 and 2,^28^,^29^ APP,^30^ the APP-binding protein Fe65,^31^ and neurexins and neuroligins.^32^ Examples of disease-associated single nucleotide polymorphisms (SNPs) are also abundant in the termini of two genes that have recently been implicated in amyotrophic lateral sclerosis (ALS): numerous mutations in the C-terminus of both TDP-43 and FUS, both involved in pre-mRNA processing and which generate inclusions in motor neurons, are reported in ALS pedigrees suggesting their causal involvement in the disease.^33^ Furthermore, usage of alternate promoters leading to inclusion of 5’ in-frame extension has been reported for example in the apoptosis regulator protein Bim in sympathetic neurons.^34^

Last, but not least, regulation of cholinesterase (ChE) levels by micro-RNA should be discussed. Over 40 different micro-RNAs are complementary to the 3’-untranslated region (UTR) of AChE mRNA, compared to 14 other micro-RNAs that are complementary to BChE’s 3’-UTR. Intriguingly, those do not overlap each other, suggesting specificity of such regulation. Also, specific ChE-targeted micro-RNAs show different evolutionary conservations and tissue distributions. We have recently shown that micro-RNA-132 arrests AChE-S mRNA translation in macrophages following inflammatory processes, thus retrieving homeostatic cholinergic signaling.^35^ Further studies will be required to explore the potential involvement of this mechanism in other diseases (e.g. neurodegeneration).

Taken together, our data demonstrate that both N- and C-terminal modulations in cholinesterases have profound roles in AD pathogenesis and that they all affect key features of AD including amyloid oligomerization, amyloid toxicity, plaque formation, tau hyperphosphorylation, apoptosis, and learning and memory impairments (Figure 3). The fact that some cholinesterase variants are protective against Aβ toxicity while others facilitate such effects offers a new possibility for therapeutic intervention. For example, cholinesterase inhibitors have been shown to up-regulate AChE-R levels by a feedback mechanism.^36^,^37^ This may explain some of the beneficial effects attributed to such inhibitors.^38^ AChE-R is also increased in the brain following stress and inflammation,^39^–^41^ possibly as a neuroprotective attempt. Further, increasing the brain levels of the neuroprotective variants by other means, for example by preventing the massive degradation of AChE-R seen in the AD brain,^11^ may help to control Aβ toxicity. The selective knock-down of specific variants using siRNA may also support efforts to specifically reduce the levels of cholinesterase variants that promote AD pathogenesis.^42^ Terminally modified ChEs are hence naturally occurring modulators of amyloid toxicity that could be harnessed in the battle against this disease.

**Figure 3:**
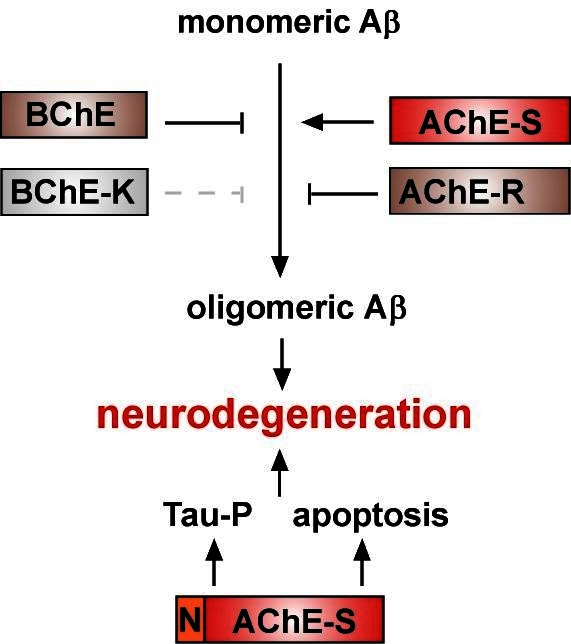
Proposed model for the combined effects of cholinesterases on Alzheimer’s disease progression. Note that both AChE-R and BChE can potentially attenuate Aβ oligomerization and its toxic effects, whereas AChE-S inversely promotes these processes, accelerating neurodegeneration. BChE-K is further impaired in its ability to attenuate fibril formation and may thus also contribute to neurodegeneration. N-AChE-S additionally promotes neurodegeneration of cholinergic neurons by the induction of apoptosis and tau hyperphosphorylation.

## Abbreviations:

Aβ: beta-amyloid;
AChE: acetylcholinesterase;
AD: Alzheimer’s disease;
ALS: amyotrophic lateral sclerosis;
APP: amyloid precursor protein;
BChE: butyrylcholinesterase;
ChE: cholinesterase;
GSK3: glycogen synthase kinase 3;
RNA: ribonucleic acid;
siRNA: small interfering RNA;
SNP: single nucleotide polymorphism;
UTR: untranslated region.
